# Smoking cessation for cancer patients through the lens of cancer specialists: challenges & solutions

**DOI:** 10.1093/eurpub/ckac131.017

**Published:** 2022-10-25

**Authors:** P Fitzpatrick, N Bhardwaj, S Syed, P Fox, K Frazer, V Niranjan, A Lyons, A McCann, S Brennan, S Guerin

**Affiliations:** 1 School of Public Health, Physiotherapy and Sports Science, University College Dublin, Dublin, Ireland; 2 Department of Preventive Medicine and Health Promotion, St. Vincent’s University Hospital, Dublin, Ireland; 3 School of Nursing, Midwifery & Health Systems, University College Dublin, Dublin, Ireland; 4 Biomolecular & Biomedical Research Institute, University College Dublin, Dublin, Ireland; 5 Department of Radiation Oncology, St. Luke’s Radiation Oncology Network, Dublin, Ireland; 6 School of Psychology, University College Dublin, Dublin, Ireland

## Abstract

**Background:**

The benefits of smoking cessation (SC) for cancer patients are widely recognised. However, there has been a limited emphasis on SC in this context and it continues to be a challenge for cancer patients. As part of a larger feasibility study aiming to develop a structured SC pathway for cancer patients in Ireland, this qualitative study explored the SC practices, experiences and opinions of oncology healthcare professionals (HCPs).

**Methods:**

Semi-structured interviews were conducted with 18 HCPs from lung, breast, cervical, head and neck and general oncology, across 4 specialist adult cancer hospitals in Ireland. Interview transcripts were analysed using thematic analysis.

**Results:**

Four key themes emerged:

(1) Frequently ask and advise but infrequently assist: most HCPs ask about smoking and many advise about available supports, but few refer patients to SC services. Where offered, referrals were to hospital SC services and/or nicotine replacement therapy was prescribed; no HCP prescribed varenicline or bupropion. Barriers included lack of time, ill-defined referral pathways and lack of knowledge.

(2) Increased willingness but differing ability to quit: most patients were interested in quitting post diagnosis and had varying support needs, linked to cancer stage, social circumstances and stress levels.

(3) Need for an integrated or parallel service: all HCPs suggested that a structured and defined referral pathway will facilitate SC.

(4) Motivational counselling and pharmacotherapy combination: many HCPs suggested face to face as the best mode of intervention initially, with regular follow ups and ongoing support virtually, started pre-treatment, with an empathetic and empowering approach with provision of both motivational counselling and SC pharmacotherapy.

**Conclusions:**

Smoking post cancer diagnosis has serious implications for cancer treatment and prognosis but is frequently overlooked. These findings will inform the design of a SC pathway for cancer patients.

**Key messages:**

• Despite increased willingness to quit, there is inadequate and inconsistent SC support provision for cancer patients.

• Tailored SC support should be an integral part of comprehensive cancer care.

